# Effects of acute ischemia and hypoxia in young and adult calsequestrin (CSQ2) knock-out and wild-type mice

**DOI:** 10.1007/s11010-022-04407-2

**Published:** 2022-03-21

**Authors:** Joachim Neumann, Konrad Bödicker, Igor B. Buchwalow, Constanze Schmidbaur, Gustavo Ramos, Stefan Frantz, Ulrich Hofmann, Ulrich Gergs

**Affiliations:** 1 Institute for Pharmacology and Toxicology, Medical Faculty, Martin Luther University Halle-Wittenberg, 06097 Halle, Germany; 2 Institute for Hematopathology, 22547 Hamburg, Germany; 3 Department of Internal Medicine and Comprehensive Heart Failure Center, University Hospital Würzburg, 97080 Würzburg, Germany; 4 Institut für Pharmakologie und Toxikologie, Martin-Luther-Universität Halle-Wittenberg, Medizinische Fakultät, Magdeburger Str. 4, 06112 Halle, Germany

**Keywords:** Calsequestrin, Hypertrophy, Aging, Ischemia, Hypoxia

## Abstract

Calsequestrin (CSQ2) is the main Ca^2+^-binding protein in the sarcoplasmic reticulum of the mammalian heart. In order to understand the function of calsequestrin better, we compared two age groups (young: 4–5 months of age versus adult: 18 months of age) of CSQ2 knock-out mice (CSQ2(−/−)) and littermate wild-type mice (CSQ2(+/+)). Using echocardiography, in adult mice, the basal left ventricular ejection fraction and the spontaneous beating rate were lower in CSQ2(−/−) compared to CSQ2(+/+). The increase in ejection fraction by β-adrenergic stimulation (intraperitoneal injection of isoproterenol) was lower in adult CSQ2(−/−) versus adult CSQ2(+/+). After hypoxia in vitro (isolated atrial preparations) by gassing the organ bath buffer with 95% N_2_, force of contraction in electrically driven left atria increased to lower values in young CSQ2(−/−) than in young CSQ2(+/+). In addition, after global ischemia and reperfusion (buffer-perfused hearts according to Langendorff; 20-min ischemia and 15-min reperfusion), the rate of tension development was higher in young CSQ2(−/−) compared to young CSQ2(+/+). Finally, we evaluated signs of inflammation (immune cells, autoantibodies, and fibrosis). However, whereas no immunological alterations were found between all investigated groups, pronounced fibrosis was found in the ventricles of adult CSQ2(−/−) compared to all other groups. We suggest that in young mice, CSQ2 is important for cardiac performance especially in isolated cardiac preparations under conditions of impaired oxygen supply, but with differences between atrium and ventricle. Lack of CSQ2 leads age dependently to fibrosis and depressed cardiac performance in echocardiographic studies.

**Supplementary Information:**

The online version contains supplementary material available at 10.1007/s11010-022-04407-2.

## Introduction

**Fig. 1:**
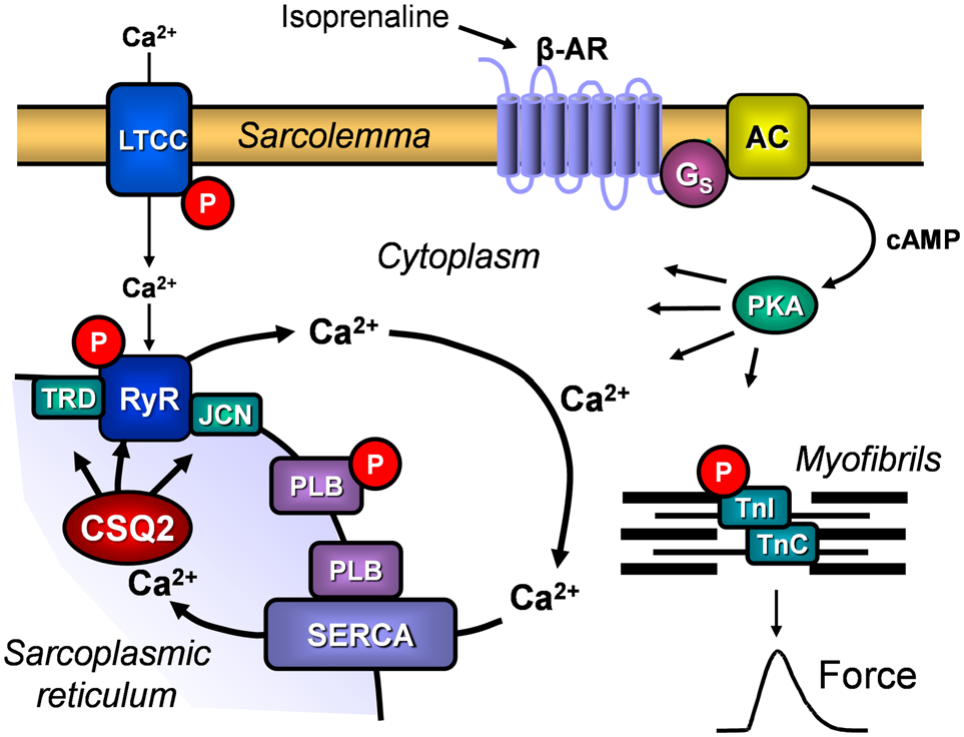
Signal transduction via β-adrenoceptors (β-AR) in cardiac myocytes and the role of the cardiac isoform of calsequestrin (CSQ2). Isoproterenol can stimulate β-AR, which, via stimulatory proteins (Gs), augment the activity of adenylyl cyclases (AC) and this leads to the formation of cAMP from ATP. Thereafter, cAMP-dependent protein kinase (PKA) leads to phosphorylation (P) and thereby to activation of the L-type calcium channel (LTCC), the ryanodine receptor (RyR, more specifically the cardiac isoform of RyR called RyR2), phospholamban (PLB), and the inhibitory subunit of troponin (TnI). Activation of LTCC leads to Ca^2+^ influx through the sarcolemma and this Ca^2+^ activates RyR, which then releases Ca^2+^ from the sarcoplasmic reticulum (SR). This released Ca^2+^ binds to myofibrils (e.g., troponin C, TnC) and leads in systole to generation of force. In diastole, Ca^2+^ is pumped via the SR Ca^2+^-ATPase (SERCA) from the cytosol into the SR, starting cardiac relaxation. Phosphorylation of TnI leads probably to a reduced Ca^2+^ sensitivity of the myofibrils and thus contributes to muscle relaxation. This Ca^2+^ in the SR binds to CSQ2 and is thereafter unable to increase force of contraction in the heart. CSQ2 can move to the vicinity of the junctional SR and there CSQ2 can interact with the proteins junctin (JCN) and triadin (TRD) and can activate RyR. The activation of RyR by CSQ2 can be altered by overexpression or deletion of triadin and junctin. Thus, junctin and triadin modify the action of CSQ2 on RyR

Ca^2+^ is crucial for excitation–contraction coupling in the mammalian heart notably the human heart. In the sarcoplasmic reticulum (SR) of the cardiomyocytes, calsequestrin is important for the storage of Ca^2+^ and the release of Ca^2+^ from the SR (more details are shown in Fig. 1; for review: [1, 2]). Calsequestrin 2 (CSQ2) is the cardiac isoform of calsequestrin coded by a different gene than the isoform CSQ1 that is found in smooth muscles but also in skeletal muscles [1, 3–5]. There are undoubtedly at least a dozen Ca^2+^-binding proteins in the heart notably calreticulin, which might compensate for CSQ2 in its function as a Ca^2+^-binding protein. However, CSQ2 that binds Ca^2+^ with high capacity is quantitatively the main Ca^2+^-binding protein in the heart [6]. Moreover, CSQ2 is hardly expressed in non-cardiomyocytes (e.g., in the esophagus or skeletal muscle), which one might interpret as indicative of a special cardiac role of CSQ2. In vitro data with recombinant proteins in artificial membranes have convincingly shown that CSQ2 can reduce the opening of the SR Ca^2+^ release channel (cardiac ryanodine receptor = RYR2) for Ca^2+^ [7, 8]. That should reduce cardiac force generation via the mechanism delineated in Fig. 1. In addition, CSQ2 might not only be relevant for physiological force generation in the heart but CSQ2 might also be important for cardiac arrhythmias: altered levels or inappropriate timing of Ca^2+^ release from the SR during diastole could induce the sarcolemmal Na^+^/ Ca^2+^ exchanger to extrude Ca^2+^ from the cytosol of the cardiomyocyte into the interstitium. Being electrogenic, an activated Na^+^/Ca^2+^ exchanger should lead to cardiac depolarization. Thus, cardiac depolarization might occur in a delayed fashion and this delayed after depolarization can bring about deadly arrhythmias.

In line with these theoretical mechanisms, several mutations in the CSQ2 gene have become known in some patients with catecholaminergic polymorphic ventricular tachycardia (CPVT) and might, at worst, lead to the death of the patients (even children) due to ventricular fibrillation [2, 9–11]. In susceptible patients, CPVT may occur for the first time after stimulation of β-adrenoceptors. This β-adrenoceptor stimulation may result from physical exercise or emotional stress [2]. An overview of incidences, manifestations, and treatment strategies for CPVT can be found elsewhere [12, 13].

Because of the putative important role of CSQ2 in cardiac force generation and in the heart beat, we ourselves and others developed and characterized homozygous CSQ2 knock-out mice (CSQ2(−/−)), using different genetic targeting strategies [14, 15]. The data of both groups are in broad agreement [14, 15]. For example, Glukhov and co-workers found interstitial fibrosis in the sinoatrial node of CSQ2(−/−) and noted altered cardiac pacemaker activity in the sinus node. They concluded that these functional and structural alterations could contribute to sinoatrial node dysfunction as well as to atrial fibrillation in patients with loss of function mutations of CSQ2(−/−) [16]. As Ca^2+^ is crucial for cardiac contractility, manipulation of cardiac CSQ2 levels might be thought to lead to altered responses to stressors that alter force of contraction by changing the Ca^2+^ homeostasis in the heart. Such stressors are cardiac hypoxia or cardiac ischemia, which we therefore studied here following established procedures in our laboratory.

Hence, we thought it worthwhile to study how fast cardiac preparations from CSQ2(−/−) mice respond with reduced contractility and enhanced arrhythmias to acute ischemia and acute hypoxia but also whether reduced force of contraction and/or arrhythmogenesis are fully reversible. To put it otherwise, we were interested how CSQ might contribute to reperfusion injury and we hypothesized that under these conditions, altered cardiac function in CSQ2(−/−) might become apparent, which might be relevant for myocardial infarction, an important clinical disease. To test these hypotheses, we used isolated cardiac preparations. Moreover, it also is known that immunological phenomena can contribute to cardiac dysfunction and even cardiac failure. This can occur in acute cardiac failure (e.g., in sepsis) or chronic heart failure. Chronic immunological activation of the heart can worsen congestive heart failure. From a mechanistically point of view, altered Ca^2+^ homeostasis can change the activity of Ca^2+^-dependent kinases and these kinases can alter the activity of transcription factors that enhance the transcription of proinflammatory genes leading to cardiac inflammation. Therefore, we hypothesized that the loss of CSQ2 might lead to immunological alterations in the heart of young and/or adult mice. Moreover, aging per se is accompanied by inflammation in many tissues, also in the heart [17]. Cardiac aging leads to functional impairment, to which fibrosis, death of cardiac cells, and subsequent exposure of antigens on cardiac cells to the immune system contribute [17–19]. For instance, in mice, CD4^+^ T cells can mediate cardiac inflammation and functional impairment upon aging [20]. In addition, cardiac aging might involve cardiac-resident macrophages [21, 22]. These hypotheses were addressed by several in vitro techniques, like flow cytometry, immunofluorescence, and assessment of fibrosis by histology. Finally, we wanted to test the hypothesis that ablation of CSQ2 might alter echocardiographic functions like left ventricular ejection fraction or left ventricular myocardial strain parameters in young as well as in adult mice.

## Materials and methods

### Mouse model

Calsequestrin (CSQ2)-deficient mice (CSQ2(−/−)), with ablation of the gene for calsequestrin 2 in the heart have been described before [14]. CSQ2(−/−) survive birth, are fertile, and show altered contractile parameters in isolated atrium or ventricle and react on stimulation with β-adrenoceptor agonists in vivo with cardiac arrhythmias [14]. Moreover, in initial data, we noted an impaired basal (that means in absence of β-adrenergic stimulation) contractility of older CSQ2(−/−) mice compared to age-matched controls [14]. These mice were used in the present study and compared with wild-type littermate mice CSQ2(+/+). We have not selected for gender. In other words, male and female mice were randomly selected. That is, in all subsequent studies, about the same number of male and female mice was included in the CSQ2(+/+) group as well as in the CSQ2(−/−) group. Table 1 gives additional information on the mice used in this study. Total number of mice were as follows: CSQ2(+/+), *n* = 38 young and *n* = 29 adult and CSQ2(−/−), *n* = 43 young and *n* = 28 adult. The numbers of mice used in the specific experiments are given in the corresponding results section. The investigation conforms to the Guide for the Care and Use of Laboratory Animals published by the National Research Council (2011) [23]. Animals were handled and maintained according to approved protocols of the animal welfare committee of the University of Halle-Wittenberg, Halle, Germany (approval reference number 42502-2-1506 MLU). The manuscript was prepared as per the ARRIVE guidelines [24].

**Table 1:** Age and gravimetric measurements of the mice studied

Genotype	CSQ2(+/+)		CSQ2(−/−)	
	Young	Adult	Young	Adult
Age (d)	139.67 ± 30.26	538.75 ± 35.64^#^	118.54 ± 12.35	458.18 ± 35.11^#^
Numbers (n)	9	12	13	11
Body weight (g)	22.56 ± 1.14	25.87 ± 1.61	21.32 ± 1.37	26.05 ± 0.79^#^
Heart weight (mg)	157.30 ± 12.19	218.69 ± 13.06^#^	174.65 ± 13.25	240.46 ± 15.58^#^
Rel. weight heart (mg/g)	6.99 ± 0.44	8.75 ± 0.61^#^	8.27 ± 0.50	8.90 ± 0.50
Rel. weight lung (mg/g)	6.98 ± 0.48	7.51 ± 0.93	8.81 ± 0.72	7.31 ± 0.29
Rel. weight liver (mg/g)	53.10 ± 1.52	51.38 ± 4.02	54.85 ± 2.64	53.96 ± 1.54
Rel. weight spleen (mg/g)	3.61 ± 0.38	4.39 ± 0.56	3.64 ± 0.28	3.93 ± 0.22
Rel. weight kidney (mg/g)	15.05 ± 0.59	15.43 ± 0.67	15.21 ± 0.42	14.35 ± 0.56

Genotype indicates CSQ2(+/+) and CSQ2(− / −) mice. Numbers of animals are indicated in the second row

^#^*p* < 0.05 vs. young age group. Relative (Rel.) weight means weight of organ divided by body weight

### Histology

Tissue samples of young and adult mouse hearts were fixed in buffered 4% formaldehyde and routinely embedded in paraffin. Four-µm-thick paraffin tissue sections were deparaffinized with xylene and grades of ethanol. For pathohistological analysis, tissue sections were routinely stained with hematoxylin/eosin, elastica van Gieson staining, and Masson–Goldner-three-color staining. For visualization and image processing, microscopic images were captured using an AxioCam digital microscope camera and the AxioVision image processing software (Carl Zeiss Microscopy, Oberkochen, Germany). The images were acquired at 96 DPI and submitted with the final revision of the manuscript at 300 DPI. Images shown are representative of at least three independent experiments, which gave similar results.

### Immunofluorescence to detect cardiac auto-reactivity

Heart-specific autoantibodies were detected by incubating histological heart slices prepared from immunoglobulin-deficient animals (AID/µS −/−) with the plasma of CSQ2(+/+) and CSQ2(−/−) animals. Next, plasma reactivity against cardiac antigens was assessed using anti-mouse IgM-Alexa 555 and anti-mouse IgG-Alexa 488 antibodies (both from Thermo Fisher Scientific, Waltham, MA, USA). As control, some heart slides were incubated with secondary antibodies only. In the absence of plasma, immunofluorescence histology analysis was performed with a Zeiss Axioskop 2 plus (Carl Zeiss Microscopy, Oberkochen, Germany) and the quantification was done with the open source software ImageJ (National Institute of Health, USA).

### Contraction experiments

Contraction experiments in isolated atrial preparations from young mice were performed as reported [23]. In brief, electrically driven (1 Hz) left atrial preparations were mounted in an organ bath. The bathing solution of the organ baths contained (in mM) NaCI, 119.8; KCI, 5.4; CaCl_2_ 1.8; MgCl_2_, 1.05; NaH_2_PO_4_, 0.42; NaHCO_3_, 22.6; Na_2_EDTA, 0.05; ascorbic acid, 0.28; and glucose, 5.05, continuously gassed with 95% O_2_ and 5% CO_2_, and maintained at 37 °C and pH 7.4. Preparations were attached to a bipolar stimulating electrode and suspended individually in 10-ml glass tissue chambers for recording isometric contractions. Force of contraction was measured with inductive force transducers connected to a digitizer (PowerLab system, ADInstruments, Oxford, United Kingdom). Contractile parameters were analyzed using Labchart 8 (ADInstruments, Oxford, United Kingdom). Each muscle was stretched to the length of maximal force of contraction. The left atrial preparations from mice were electrically stimulated at 1 Hz with rectangular pulses of 5-ms duration; the voltage was ~ 10–20% greater than threshold. Contractile parameters were continuously evaluated by the software used. Force of contraction was defined as the difference between maximum and minimum tension at constant muscle length. Spontaneously beating right atrial preparations from mice were used to study any chronotropic effects. After an equilibration time of 30 min, buffer was bubbled with a mixture of 95% N_2_ and 5% CO_2_, in order to induce hypoxia. Under these conditions, the atrium was maintained at 37 °C and pH at 7.4. The organ bath contained double glass walls allowing pre-warmed water to circulate and this allowed us to maintain 37 °C in the organ bath before, during, and after hypoxia. This protocol was successfully used and published previously by our group [25–27].

Contraction experiments in isolated whole-perfused hearts from young mice were also performed using established protocols in our laboratory [26, 27] with modifications of the original description [28]. In brief, spontaneously beating hearts were retrogradely perfused under constant flow (2 ml/min) and the force of contraction was measured mechanically at the apex of the heart by means of a force transducer. This signal was digitized as described above for atrial preparation. After 20 min of equilibration, hypoxia was induced for 20 min by stop of the perfusion pump followed by reperfusion by starting the perfusion pump again for 15 min. Contractile parameters were continuously electronically stored. Before stop of perfusion (basal parameters), at the end of stop of perfusion, and after reperfusion (hypoxia parameters) force parameters were measured and subjected to statistical analysis. The hearts remained at room temperature during this global ischemia and were not warmed. Thereafter, the pump was started again to simulate cardiac reperfusion.

### Flow cytometry

A cell suspension of whole-heart samples was obtained after collagenase type II digestion (1.000 IU/mL. 37 °C. 30 min) and then ground in Hanks’ balanced salt solution containing 1% (wt/vol) BSA (BSS/BSA) using a 40-μM cell strainer, according to previous descriptions [29]. Lymph node samples were ground and prepared in BSS/BSA [5% (vol/vol) FCS]. Cells were stained using the following fluorescently labeled antibodies (in different combinations): anti-CD4 (clone RM4-5), anti-CD8 (clone 53–6.7), anti-CD44 (clone IM7), anti-CD45 (clone 30-F11), anti-CD45/B220 (clone RA3-6B2), and anti-TCRβ (clone H57-597). All antibodies were purchased from BioLegend (BioLegend GmbH, Koblenz, Germany). Measurements were made using an LSR-Fortessa cell analyzer (Becton Dickinson GmbH, Heidelberg, Germany) and the data were analyzed using FlowJo Single-Cell Analysis Software v10 (FlowJo LLC, Ashland, OR, USA).

### Echocardiography

Transthoracic echocardiographic measurements were performed in spontaneously breathing young and adult mice anaesthetized with 1.5% isoflurane using a Vevo 2100 system equipped with a MS 550D transducer (Visual Sonics, Toronto, Canada). Analysis of the myocardial wall motion was performed using the VisualSonics VevoStrain™ Software (Visual Sonics, Toronto, Canada) following the instructions of the manufacturer. After induction of anesthesia the mice were positioned on a 37-°C heating pad. Two-dimensional images and M-mode tracings from the parasternal long-axis view were recorded. Cardiac dimensions were measured and the ejection fraction of the hearts was calculated. Isoproterenol was injected, where indicated, at 1 mM in 100 µl, as reported before [30].

### Data analysis

Data shown are means ± SEM. Statistical significance was estimated using *T* tests or for multiple comparisons by analysis of variance followed by Bonferroni’s two-sided *t* test. A *p*-value < 0.05 was considered significant.

### Drugs and materials

Isoproterenol was obtained from Sigma-Aldrich (Taufkirchen, Germany). All other chemicals were of analytical grade. Deionized water was used throughout the experiments. Stock solutions were freshly prepared daily.

## Results

### Gravimetric results

In young mice, body weights were similar in CSQ2(−/−) or littermate wild-type mice CSQ2(+/+) (Table 1). However, the relative heart weights (heart weight divided by body weight) were lower in young CSQ2(−/−) than in littermate young CSQ2(+/+) mice (Table 1). Aging increased heart weight in CSQ2(−/−) and CSQ2(+/+) (Table 1). Absolute body weights were larger in adult KO mice than in adult CSQ2(+/+) mice (Table 1). In contrast, relative heart weights were increased in adult CSQ2(+/+) but not adult CSQ2(−/−) (Table 1). As a control, we determined also relative spleen, kidneys, liver, and lung weights, which were not significantly different between genotypes or ages (Table 1).

### Basal echocardiography

Next, echocardiographic measurements in narcotized animals were performed in order to study cardiac function in vivo. It is noteworthy that the ejection fractions under basal conditions were not different between genotypes in young mice, whereas in adult CSQ2(−/−) mice, the basal ejection fraction as well as the beating rates were lower than in adult CSQ2(+/+) mice, arguing for an age-dependent reduction in left cardiac ejection fraction due to CSQ2 ablation (Table 2) which confirms an earlier study [12] on a different group of animals from our lab. Here, we extend those data by a rather complete assessment of noninvasive echocardiographic data, notably flow measurements of vessels, functional assessment of the mitral valve, tissue Doppler of the left ventricle, and septal and left ventricular systolic and diastolic diameters.

**Table 2:** Echocardiographic measurements in anesthetized mice

Echocardiographic Parameters	Age	Stimulation	Genotype				Numbers (*n*)
			CSQ2(+/+)		CSQ2(−/−)		
			Mean	SEM	Mean	SEM	
Aorta descendens peak velocity (mm/s)	Adult	Ctr	950.61	28.31	906.12	60.21	8/7
		Iso	1,017.67	58.26	1,144.29^+#^	49.97	3/4
	Young	Ctr	920.36	34.46	960.36	57.97	6/7
		Iso	x	x	880.15	23.90	0/2
Aorta descendens VTI (mm)	Adult	Ctr	29.27	1.35	27.18^#^	1.30	8/7
		Iso	25.41	2.03	25.91	1.65	3/4
	Young	Ctr	29.77	1.71	32.23	1.68	6/7
		Iso	x	x	28.09	0.98	0/2
Pulmonary artery peak velocity (mm/s)	Adult	Ctr	711.40	31.94	591.18	56.14	8/7
		Iso	845.89	57.70	879.39^+^	29.06	3/4
	Young	Ctr	794.85	28.79	688.91*	32.29	6/7
		Iso	794.98	49.45	838.00^+^	34.40	6/7
Pulmonary artery VTI (mm)	Adult	Ctr	25.79	0.67	20.68*^#^	1.08	8/7
		Iso	22.20	2.54	23.89	0.85	3/4
	Young	Ctr	29.92	1.08	26.30	1.10	6/7
		Iso	25.17	1.69	25.40	1.15	6/7
Ejection fraction	Adult	Ctr	76.46	1.99	62.51*	4.26	8/7
		Iso	90.82^+^	1.61	71.75*^#^	2.89	8/7
	Young	Ctr	78.77	1.85	74.50	4.15	6/7
		Iso	90.75^+^	2.62	89.51^+^	2.60	6/7
Heart rate (BPM)	Adult	Ctr	496.72	9.79	432.90*	32.07	8/7
		Iso	550.56^+^	4.41	532.40^+^	14.17	8/7
	Young	Ctr	502.26	26.66	421.65	30.68	6/7
		Iso	557.75	34.41	554.81^+^	16.86	6/7
Left ventricular end-diastolic diameter (mm)	Adult	Ctr	3.22	0.16	3.31	0.13	8/6
		Iso	2.78	0.12	2.90^+^	0.10	6/6
	Young	Ctr	2.99	0.11	3.15	0.19	6/7
		Iso	2.54	0.21	2.54	0.21	6/7
Left ventricular end-systolic diameter (mm)	Adult	Ctr	1.62	0.05	2.18*	0.13	6/7
		Iso	0.96^+^	0.09	1.59*^+#^	0.11	6/7
	Young	Ctr	1.63	0.07	1.82	0.20	6/7
		Iso	0.95^+^	0.16	1.05^+^	0.17	6/7

Genotype indicates CSQ2(+/+) and CSQ2(−/−) mice. Tissue *E*ʹ/*A*ʹ indicates tissue Doppler data where the *E*ʹ-value is divided by the *A*ʹ- value. Adult means mice about 18 months old and young means about six months old. See Table 1 for exact ages. Numbers of animals are indicated in the last column. X = no stable echocardiographic recordings could be obtained. Here, only parameters with differences between genotypes are shown. For the complete data table see supplementary table 1

^*^p < 0.05 vs. CSQ2(+/+)

^#^p < 0.05 vs. young age group of each genotype

^+^*p* < 0.05 vs. control (Ctr). Ctr means control condition that is before β-adrenergic stimulation by injection of isoproterenol. Iso means values after injection of isoproterenol

Here, it is noteworthy that under basal conditions the velocity time integral (VTI) through the aorta descendens was lower in adult CSQ2(−/−) compared to young CSQ2(−/−), suggesting an involvement of aging. In adult CSQ2(−/−) the VTI through the pulmonary artery was lower than in young CSQ2 (−/−) but notably also than in adult CSQ2(+/+) suggesting both, an involvement of aging and gene ablation. Moreover, left ventricular end-systolic diameter was larger in adult CSQ2(−/−) than in adult CSQ2(+/+), indicative of a role of CSQ2 knock-out. Furthermore, the wall motion was assessed by regional strain parameters using under these basal echocardiographic conditions (Table 3). Of note, effects of genotype and gene ablation behaved in some parameters differently. For instance, radial lateral wall strain increased with aging in CSQ2(−/−) as well as in CSQ2(+/+) (Table 3). Mid longitudinal posterior strain decreased with aging in CSQ2(+/+) but increased with aging in CSQ2(−/−). Mid longitudinal posterior strain rates were higher in adult CSQ2(−/−) than in young CSQ2(−/−). However, mid longitudinal posterior strains and mid longitudinal strain rates were lower in CSQ2(−/−) than in CSQ2(+/+) (Table 3).

**Table 3:** Basal strain parameters

Echocardiographic Parameters	Age	Genotype				Numbers (*n*)
		CSQ2(+/+)		CSQ2(−/−)		
		Mean	SEM	Mean	SEM	
Strain radial lateral wall (%)	Adult	36.88^#^	3.07	32.91^#^	8.22	4/4
	Young	14.54	4.41	11.21	2.31	6/7
Strain longitudinal posterior mid (%)	Adult	13.15^#^	1.33	16.5^#^	1.74	8/8
	Young	22.20	2.63	10.56*	1.79	6/7
Strain rate longitudinal posterior mid (1/s)	Adult	6.86	0.72	7.49^#^	0.87	8/8
	Young	8.46	1.15	4.87*	0.41	6/7
Strain rate circumferential lateral wall (1/s)	Adult	16.14	3.52	4.12	3.82	4/4
	Young	10.88	0.67	11.33	1.18	6/7

Using echocardiography, the strain of regions of the heart assessed by B-mode measurements using the software supplied for the Visual Sonics system Vevo 2100. Genotype indicates CSQ2(+/+) and CSQ2(− / −) mice. Basal means control condition that is before β-adrenergic stimulation by injection of isoproterenol. Adult means mice about 18 months old and young means about 6 months old. See Table 1 for exact ages. Numbers of animals are indicated in the last column. Here, only parameters with differences between genotypes are shown. For the complete data table see Supplementary Table 2

^*^p < 0.05 vs. CSQ2(+/+)

^#^*p* < 0.05 vs. young age group of each genotype

### Echocardiography under isoproterenol

Next we assessed the response to an intraperitoneal injection of isoproterenol, which stimulates β-adrenoceptors, in a dose that we have used before in echocardiographic studies of the heart of other transgenic mouse lines [30, 31]. Isoproterenol led to higher peak velocity in the aorta descendens of adult versus young CSQ2(−/−). Interestingly, while injected isoproterenol increased ejection fraction, in adult and young mice both transgenic and CSQ2(+/+), and the percentile increase in injection fraction was smaller in adult (but not young) CSQ2(−/−) compared to adult CSQ2(+/+) (Table 2). After injection of isoproterenol, the left ventricular end-systolic diameters were diminished in all genotypes; but less so in adult CSQ2(−/−) compared to adult CSQ2(+/+), in line with the findings on ejection fraction and suggesting a gene knock-specific effect of aging (Table 2). In addition, supposedly as a consequence of impaired stimulation of left ventricular function by injection of isoproterenol, likewise the pulmonary artery velocity time integral exhibited lower values in CSQ2(−/−) compared to CSQ2(+/+) after injection of isoproterenol (Table 2).

### Fibrosis

**Fig. 2:**
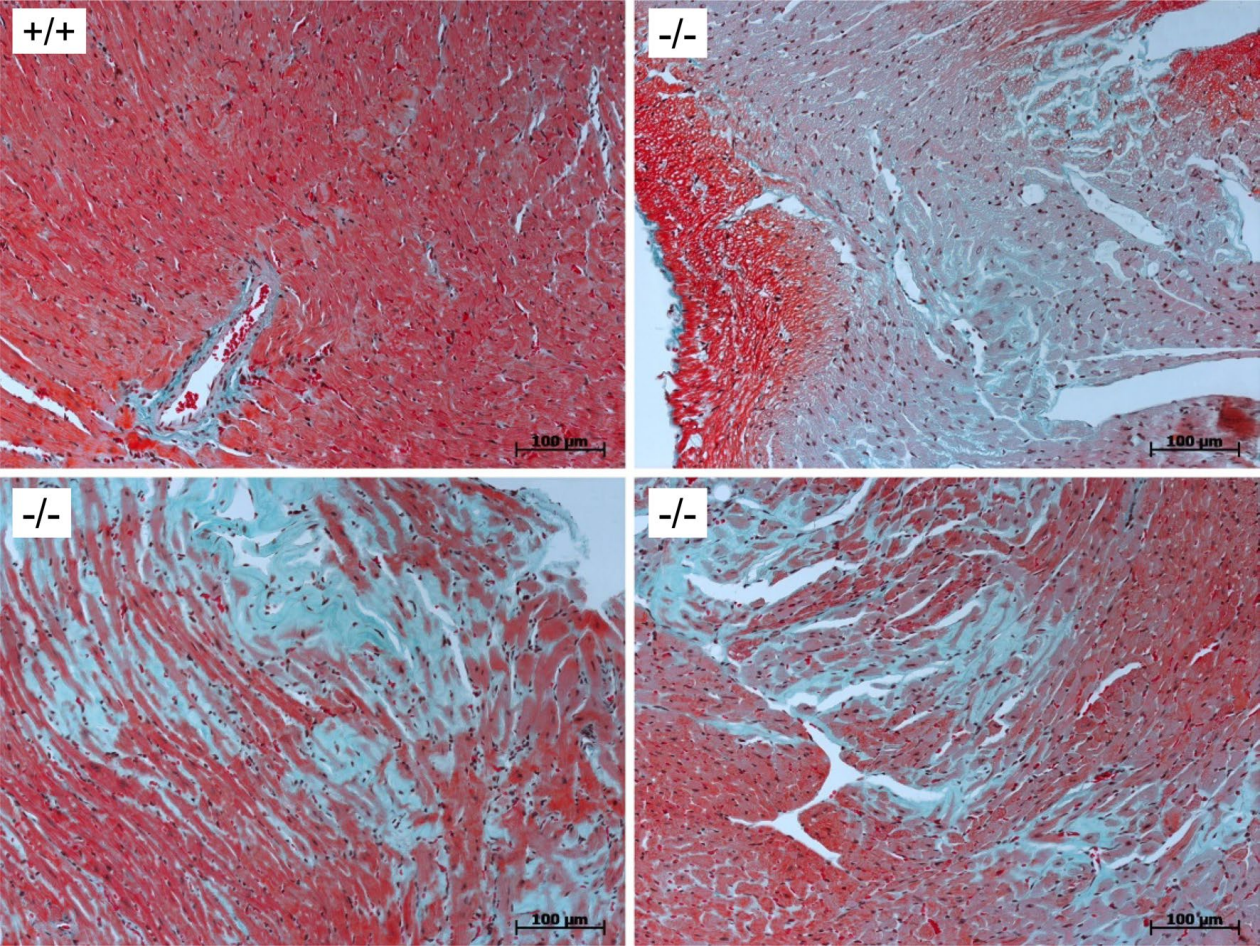
Histology of Masson–Goldner staining of ventricular slices from CSQ2(+/+) and CSQ2(−/−) mice. The magnification is given by the horizontal size markers in µm

We tested the hypothesis that upon aging, fibrosis might be detectable histologically in adult CSQ2(−/−). Employing Masson–Goldner three-color staining, we found a heavy fibrosis in the ventricles (n = 3) from adult CSQ2(−/−) but no signs of fibrosis in the ventricles (n = 3) from adult CSQ2(+/+) (Fig. 2). We display here (Fig. 2) only one of three adult CSQ2(+/+) but failed to detect fibrosis in the other samples from adult CSQ2(+/+). We failed to detect fibrosis histologically in samples from young mice of CSQ2(−/−) and CSQ2(+/+) (n = 3 each, data not shown).

### Immune responses

**Fig. 3:**
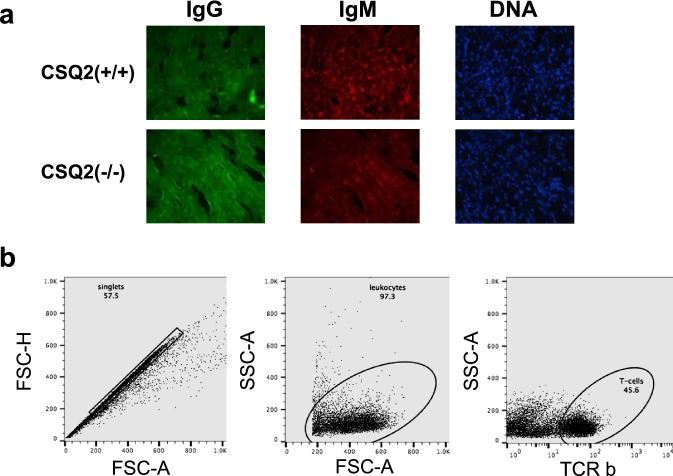
**a** Representative immunofluorescence images of heart preparations from CSQ2(+/+) and CSQ2(−/−) incubated with anti-IgG (green) and anti-IgM (red) monoclonal antibodies. Nuclei (DNA) were counterstained with DAPI (blue). **b** FACS gating on T cells

In addition, we sought to assess whether immune-mediated responses could contribute to the cardiac decline observed in CSQ2(−/−). Therefore, we performed flow cytometry analyses to assess the distribution of all major lymphocyte subsets in collagenase-digested hearts and mediastinal lymph nodes (heart -draining) obtained from CSQ2(+/+) and CSQ2(−/−). As shown in Fig. 3 and Table 4, we did not observe any differences in the composition of cardiac T- and B-lymphocytes subsets across different genotypes, indicating that in situ inflammation might not contribute to CSQ2-mediated cardiac decline. Nevertheless, CSQ2(−/−) presented an enlargement of the heart-draining lymph nodes, especially associated with an expansion of the B-cell compartment (Fig. 3, Table 4). These alterations in the B-cell compartment led us considering a possible role for autoantibody-mediated cardiac damage in CSQ2(−/−). To assess the putative role of autoantibodies in this model, we incubated plasma obtained from CSQ2(+/+)- and CSQ2-deficient mice with heart slices obtained from immunoglobulin-deficient mice. The putative presence of heart-reactive antibodies in the sera of CSQ2(+/+) versus CSQ2(−/−) was then detected using an anti-mouse IgG and IgM conjugated with fluorescent dyes, followed by inspection in the fluorescence microscope. As shown in Fig. 3 and summarized in Table 4, the plasma obtained from CSQ2(+/+) and CSQ2(−/−) showed the same cardiac autoantibody pattern. These data suggest that autoantibodies are not likely to play a role in the CSQ2-mediated cardiac functional decline.

**Table 4:** Immunological parameters

Immune parameters					
Age	Young		Adult		Numbers of mice
Genotype	CSQ2(+/+)	CSQ2(−/−)	CSQ2(+/+)	CSQ2(−/−)	Young; Adult
FACS					
Mediastinal Lymph Nodes					
cells/LN	114,756 ± 26,300	256,597 ± 118,998	58,846 ± 20,507	257,067 ± 94,118	3/3; 6/5
Frequency of B cells	24 ± 4.73	30 ± 3.82	26 ± 4.32	38 ± 5.08	3/3; 6/5
Frequency of CD 4 + CD44 + cells	10 ± 4.14	12 ± 1.37	43 ± 3.56^#^	31 ± 5.36	3/3; 6/5
Frequency of CD 8 + CD44 + cells	11 ± 3.93	11 ± 1.41	55 ± 11.71^#^	41 ± 13.24	3/3; 6/5
Heart					
Frequency of T cells	27.4 ± 1.11	21.1 ± 1.56	7.91 ± 1.94^#^	10.48 ± 3.36	3/3; 6/5
Frequency of B cells	32 ± 2.04	27.67 ± 2.46	11.96 ± 4.73^#^	12.39 ± 3.01^#^	3/3; 5/5
Immunofluorescence monoclonal antibodies					
Anti-IgM	22,455 ± 4,457	18,800 ± 2,878	34,057 ± 5,158	29,300 ± 4,565	6/5; 3/5
Anti-IgG	19,808 ± 3,066	25,407 ± 1,339	26,923 ± 2,559	25,192 ± 2,409	6/5; 3/5
DAPI	91,735 ± 6,582	76,384 ± 12,454	67,490 ± 9,090	63,082 ± 10,702	6/5; 3/5

Using fluorescence-activated cell sorting (FACS) with FlowJo Single-Cell Analysis Software leucocytes in the samples were analyzed. Mean intensity of immunofluorescence signals using an anti-mouse IgG, IgM, and DAPI conjugated with fluorescent dyes was measured using a Zeiss Axioskop 2 Plus and ImageJ software. Genotype indicates CSQ2(+/+) and CSQ2(−/−) mice. Adult means mice about 18 months old and young means about six months old. See Table 1 for exact ages. Numbers of animals are indicated in the last column

^#^*p* < 0.05 vs. young age group of each genotype. *FACS* fluorescence-activated cell sorting, *LN* lymph nodes, *Anti-IgM* anti-immunoglobulin M antibody, *Anti-IgG* anti-immunoglobulin G antibody, *DAPI* 2-[4-(aminoiminomethyl)phenyl]-1*H*iIndole-6-carboximidamide hydrochloride (for labeling of nuclei)

### Hypoxia

As a further stressor for cardiac function in young CSQ2(−/−), we induced hypoxia in paced isolated left and spontaneously beating right atrial preparations by replacing oxygen with nitrogen in the organ bath. We have used this method successfully in other transgenic mice to mimic hypoxia in vivo [26, 27]. This hypoxia in the organ bath was able to reduce force of contraction in the left atrial preparations within 30 min nearly completely, consistent with our earlier publications [31]. Thereafter, normoxia was re-instated but developed force of contraction and its first derivative did not return to the values observed before hypoxia, in both CSQ2(+/+) and CSQ2(−/−) (data not shown). Of note, at 10 min of reperfusion, the decrease in developed force of contraction (expressed in percentage of pre-hypoxic values) and its first derivative with regard to time were more pronounced (that is after previous hypoxia) in CSQ2(−/−) than in age-matched CSQ2(+/+): 28.1 ± 5.92% of pre-hypoxic levels versus 10.1 ± 1.59% of pre-hypoxic values or 34.8 ± 10.2 mN/sec and 17.2 ± 2.89 mN/sec, respectively (p < 0.05; n = 8–9), suggesting that CSQ2 is important for the mouse atrium to sustain better short-term hypoxia. Time to peak tension was unchanged in hypoxia in both genotypes. However, time of relaxation was prolonged in both, CSQ2(+/+) and CSQ2(−/−), under hypoxia and under re-oxygenation, it was normalized only in CSQ2(+/+) but in CSQ2(−/−), and time of relaxation fell below the pre-hypoxic values: 33.9 ± 1.75 ms versus 30.0 ± 1.68 ms (*p* < 0.05; *n* = 8–9).

Heart rates in spontaneously beating right atrial preparations were increased at the beginning of hypoxia and all preparations of CSQ2(−/−) and CSQ2(+/+) went into arrhythmias at the end of the maximum time of hypoxia, but returned to sinus rhythm and pre-hypoxia rates. Heart rates in CSQ2(+/+) and CSQ2(−/−) remained below pre-hypoxic heart rate (data not shown). Thus, no difference with respect to heart rate were notable in isolated right atria between the genotypes studied.

### Ischemia

Next, we studied cardiac ischemia in an in vitro model as a further stressor. Here, in order to assess left ventricular function, protocols were used that have been published before [26, 27]. We studied global ischemia in the isolated spontaneously beating retrogradely perfused mouse heart (Langendorff heart, [28]): after stabilization of the contractile function in the isolated perfused hearts for 30 min, global ischemia was induced by stopping perfusion of the heart and this rapidly led to a cessation of contraction. The time to the stop of any heart beat amounted to 65 ± 7.3 s and 145 ± 15 s in young CSQ2(−/−) and CSQ2(+/+), respectively (*n* = 5, *p* < 0.05), suggesting a protective role of CSQ2. Reperfusion led to the reappearance of spontaneous beating of the hearts of both genotypes. After 15 min of reperfusion, developed tension was lower in CSQ2(−/−) than in CSQ2(+/+) (15.6 ± 3.23 mN and 25.8 ± 2.3) but also its first derivate versus time was lower in CSQ2(−/−) than in CSQ2(+/+) (dF/dt: 458 ± 81.7 mN/s and 776 ± 62.1 mN/s; *n* = 5 each, *p* < 0.05), suggesting that CSQ2 is possibly detrimental for maintenance of left ventricular force after global ischemia.

From these data one could argue that CSQ2 is relevant in cardiac hypoxia and aging in a mouse model. Data in patients are awaited with interest.

## Discussion

Novel findings in this report include a systematic comparison of echocardiographic parameters of CSQ2(−/−) with age-matched controls. This was done with mice at younger and older age. Moreover, we present new data on ventricular fibrosis as a possible mechanism that might explain some of the echo findings in older CSQ2(−/−). Another new finding in this report is that CSQ2(−/−) are less able to withstand the stress of hypoxia and ischemia under the present experimental conditions.

We had deleted constitutionally the coding region of exon [14, 32] and, using classical methods, we finally achieved one heterozygous founder, the offspring of which was used also in the present studies. In the initial description of this mouse, we reported the absence of CSQ2 on protein level in Western blots of atria and ventricles from CSQ2(−/−), implying successful deletion of CSQ2 [14]. The observation that homozygous CSQ2(−/−) were viable and fertile, most likely means that CSQ2 is not absolutely required for Ca^2+^ storage and handling in the mammalian heart. This suggests that the cardiac function of CSQ2 must be more subtle than originally assumed from biochemical studies: the function of CSQ2 as a Ca^2+^ buffer is apparently overtaken, at least in part, by other proteins, which act as Ca^2+^ stores in the SR. However, it needs to be elucidated how other proteins can compensate, exempli gratia, for the modulatory role of CSQ2 on RYR2 function. Structurally, in accordance with patient data and animal data [15, 33], the hearts at half a year of age from CSQ2(−/−) were normal based on gross inspection, echocardiography and HE staining. But the increased left atrial weights of CSQ2(−/−) at half a year of age which we reported previously [14] suggested to us a beginning heart disease and motivated us to study older (adult) CSQ2(−/−) in the present work. We hypothesized that older CSQ2(−/−) might develop cardiac hypertrophy and signs of heart failure which might be explained by an increased diastolic Ca^2+^ due to impaired SR Ca^2+^ release. An increase in diastolic Ca^2+^ can lead not only to arrhythmias but also activates Ca^2+^ kinases and thus induces gene transcription programs leading to cardiac hypertrophy and heart failure (overview in [34]). In agreement with other groups, we found bradycardia in CSQ2(−/−) in ECG [15, 35]. The bradycardia might indicate a role of CSQ2 in the cardiac clock, the pacemaker of the heart, which is known to be tightly linked to SR Ca^2+^ release [34]. The fact that we noted reduced beating rates in living adult animals is plausible based on the literature: others noted a tendency (not reaching *p* < 0.05) in 12-month-adult CSQ2(−/−) for reduced beating rate. Our interpretation would be that we measured at a later time point (18 months) and as bradycardia increased with age, the difference finally gained significance. We ourselves in this report as well as in earlier work [14] as others before [15] did not detect altered basal contractility in echocardiography (using ejection fraction as parameter of left ventricular systolic function in vivo) in young CSQ2(−/−) suggesting to us that at this age any detrimental effects of deletion of CSQ2 are still effectively compensated by other proteins. However, in older CSQ2(−/−), cardiac function was impaired as demonstrated by a lower ejection fraction, an increased left ventricular end-systolic diameter, and a partially impaired arterial flow, suggesting that either the compensatory mechanisms were overwhelmed with time or that additional detrimental factors, like fibrosis emerge (see below). It needs some comment that the effect of β-adrenergic stimulation on ejection fraction by isoproterenol was smaller in adult CSQ2(−/−) (Table 2). This is not simply a result of aging, because the effect of isoproterenol was intact in age-matched CSQ2(+/+). Thus, specific alterations in the β-adrenergic signal transduction system (Fig. 1) are probably present, which need to be defined. Moreover, the fibrosis, which we observed, might contribute to the impaired contractile response to isoproterenol. In addition, this fibrosis might also explain, at least in part, why we measured altered strain values (Table 3) in older CSQ2(−/−): strain is regarded as being correlated with relaxation and diastolic function which is known to be impaired in fibrosis for instance in patient who survived a myocardial infarction.

The role of CSQ2 in hypoxia and ischemia is at present somewhat controversial. There are conflicting reports, whether the expression of the CSQ2 protein is altered in hypoxia or ischemia in the heart. This seems to be model-dependent: it is important, which species is studied (rabbit; rat; mouse; human), how long the lack of oxygen and how long the reperfusion lasted, and whether cells or atrial preparations or whole hearts were studied. Most reports failed to detect alterations in the protein expression of CSQ2 in the heart due to ischemia or hypoxia [36–38]. However, others reported reduction of the expression of CSQ2 in acute hypoxia in neonatal rat cardiomyocytes [39] or an increase in isolated rat hearts after ischemia and reperfusion [40].

We would like to point out that we observed in the present report opposite effects of CSQ2 deletion on response to hypoxia in atrium versus ventricle, at least in the mouse. The fact that we present evidence for a detrimental effect of CSQ2 in the left ventricle and beneficial effects of CSQ2 in atrium might be related to the observation that the Ca^2+^ handling is different in atrium versus ventricle in the mammalian heart. Anatomically, it is well established that the SR is larger and much more developed in the mammalian ventricle than in the mammalian atrium [34]. Furthermore, excitation–contraction coupling shows functional regional differences: in the mouse atrium, an increase in the beating rate will reduce force of contraction, whereas in the isolated mouse ventricular preparation, an increase in the beating rate will increase force (negative and positive “treppe” or staircase phenomena) [14, 34]. In addition, another difference between atrium and ventricle lies in the observation that the sarcolemmal Ca^2+^ flow is thought to be more important in atrium than ventricle of the mouse and rat to initiate excitation–contraction coupling [34]. How exactly CSQ2 would alter Ca^2+^ handling in hypoxic atrial cells versus ventricular cells would be important to study in the future. Moreover, it needs to be confirmed that these regional differences occur also in carriers of loss of function mutations of CSQ2. We would predict a similar pattern in these patients.

It is currently unclear, why we noted fibrosis in CSQ2(−/−) ventricles. However, these findings are consistent with reports of others that fibrosis occurs in the pacemaker region in three- and twelve-month adult CSQ2(−/−) hearts [16]. They suggested that the increased basal cytosolic Ca^2+^ levels in CSQ2(−/−) might activate Ca^2+^-dependent enzymes that promote gene transcription of pro-fibrotic proteins. The same arguments could be made for the ventricular fibrosis that we report here. But this is just speculative as strong evidences are still missing. Alternatively, they speculated that the higher incidence of arrhythmias in CSQ2(−/−) led to arrhythmia-induced hypertrophy [16]. The fibrosis noted in this study may contribute to the impaired ejection fraction in the 18-month-old CSQ2(−/−), which we report here.

Meanwhile, it is accepted that cardiovascular diseases are associated with cardiovascular inflammation. This includes ischemic heart diseases like myocardial infarction where pro-inflammatory macrophages release pro-inflammatory cytokines and other immune cells, like T cells, for example, CD8 + and CD4 + T cells are involved [41]. But also hypoxia and reperfusion lead to cytokine release [41]. Moreover, cardiac hypertrophy as well as aging is associated with inflammation processes that are involved in the progress of non-ischemic heart failure [41]. In wild-type C57BL/6 J mice (the same genetic background as the CSQ2(−/−) mice), it was noted that anti-inflammatory macrophages decrease with age, whereas pro-inflammatory macrophages increase with age in the heart [42]. Others found that in cardiac aging of C57BL/6 J mice, the number of macrophages declined from 2–3 months to 12–15 months, whereas the number of granulocytes in the heart increased [20]. This was accompanied by a decline in fractional shortening and increased end-diastolic anterior wall thickness [20]. In cardiac lymph nodes, increased inflammation was seen upon aging because the number of CD4 + T cells rose [20]. Therefore, we expected that also in CSQ2(−/−) hearts, at least in the adult group, signs of inflammation should be visible because of the hypertrophy, fibrosis, and impaired cardiac function. But contrary to our expectations, we could not found indications to cardiac inflammation. In addition, one could ask whether calsequestrin was also present in blood cells like B cells and might be affected by our general knock-out of CSQ2 and might explain hypertrophy in the aging CSQ2(−/−) hearts. However, to the best of our knowledge, in blood cells only CSQ1 is present, which is coded by a different gene and that should not be altered in our CSQ2-deficient mice [14, 43, 44].

Limitations: a direct link between CSQ2 and fibrosis is missing and remains to be established. A direct mechanism starting in fibrocytes appears unlikely given the observation by others that cardiac fibrocytes do not express CSQ2 [4]. Therefore, indirect mechanisms like cardiac hypertrophy or pro-fibrotic mediators produced by the CSQ2(−/−) cardiomyocytes have to be considered. However, in future work it would be informative to measure intracellular Ca^2+^ transients in cardiomyocytes from younger and older CSQ2(−/−) (also in hypoxia) and the effects of isoproterenol to these transients. Moreover, the biochemical interaction of RYR2 with triadin and junctin in younger and older CSQ2(−/−) should be studied. This could be combined with the study of ryanodine on intracellular Ca^2+^ transients in cardiomyocytes of these mice, which was regrettably beyond the scope of the present paper.

In summary, the present data argue: CSQ2 is relevant in cardiac hypoxia and aging in the mammalian heart. Data in patients with functional ablation of CSQ2 are awaited with interest. The present work might motivate careful study of patients with functional loss of CSQ2 with the questions in mind, whether these patients are more likely to develop impaired ejection fraction in echocardiography than control patients and this information might be clinically helpful for risk stratification.

## Supplementary Information

Below is the link to the electronic supplementary material.

Supplementary file1 (DOCX 25 kb)

## Data Availability

Data are available on reasonable request.

## References

[1] Kornyeyev D, Petrosky AD, Zepeda B (2012). Calsequestrin 2 deletion shortens the refractoriness of Ca2+ release and reduces rate-dependent Ca2+-alternans in intact mouse hearts. J Mol Cell Cardiol.

[2] Priori SG, Chen SRW (2011). Inherited dysfunction of sarcoplasmic reticulum Ca2+ handling and arrhythmogenesis. Circ Res.

[3] Zhang L, Kelley J, Schmeisser G (1997). Complex formation between junctin, triadin, calsequestrin, and the ryanodine receptor. Proteins of the cardiac junctional sarcoplasmic reticulum membrane. J Biol Chem.

[4] Jones LR, Suzuki YJ, Wang W (1998). Regulation of Ca2+ signaling in transgenic mouse cardiac myocytes overexpressing calsequestrin. J Clin Invest.

[5] McFarland TP, Milstein ML, Cala SE (2010). Rough endoplasmic reticulum to junctional sarcoplasmic reticulum trafficking of calsequestrin in adult cardiomyocytes. J Mol Cell Cardiol.

[6] MacLennan DH, Wong PT (1971). Isolation of a calcium-sequestering protein from sarcoplasmic reticulum. Proc Natl Acad Sci USA.

[7] Györke I, Hester N, Jones LR (2004). The role of Calsequestrin, Triadin, and Junctin in conferring cardiac ryanodine receptor responsiveness to luminal calcium. Biophys J.

[8] Györke S, Terentyev D (2008). Modulation of ryanodine receptor by luminal calcium and accessory proteins in health and cardiac disease. Cardiovasc Res.

[9] Lahat H, Pras E, Olender T (2001). A missense mutation in a highly conserved region of CASQ2 is associated with autosomal recessive catecholamine-induced polymorphic ventricular tachycardia in Bedouin families from Israel. Am J Hum Genet.

[10] Postma AV, Denjoy I, Kamblock J (2005). Catecholaminergic polymorphic ventricular tachycardia: RYR2 mutations, bradycardia, and follow up of the patients. J Med Genet.

[11] Di Barletta MR, Viatchenko-Karpinski S, Nori A (2006). Clinical phenotype and functional characterization of CASQ2 mutations associated with catecholaminergic polymorphic ventricular tachycardia. Circulation.

[12] Viskin S, Belhassen B (1998). Polymorphic ventricular tachyarrhythmias in the absence of organic heart disease: classification, differential diagnosis, and implications for therapy. Prog Cardiovasc Dis.

[13] Kallas D, Lamba A, Roston TM (2021). Pediatric catecholaminergic polymorphic ventricular tachycardia: a translational perspective for the clinician-scientist. Int J Mol Sci.

[14] Gergs U, Fahrion CM, Bock P (2017). Evidence for a functional role of calsequestrin 2 in mouse atrium. Acta Physiol (Oxf).

[15] Knollmann BC, Chopra N, Hlaing T (2006). Casq2 deletion causes sarcoplasmic reticulum volume increase, premature Ca2+ release, and catecholaminergic polymorphic ventricular tachycardia. J Clin Invest.

[16] Glukhov AV, Kalyanasundaram A, Lou Q (2015). Calsequestrin 2 deletion causes sinoatrial node dysfunction and atrial arrhythmias associated with altered sarcoplasmic reticulum calcium cycling and degenerative fibrosis within the mouse atrial pacemaker complex1. Eur Heart J.

[17] Yu Q, Watson RR, Marchalonis JJ (2005). A role for T lymphocytes in mediating cardiac diastolic function. Am J Physiol Heart Circ Physiol.

[18] Gurujeyalakshmi G, Giri SN (1995). Molecular mechanisms of antifibrotic effect of interferon gamma in bleomycin-mouse model of lung fibrosis: downregulation of TGF-beta and procollagen I and III gene expression. Exp Lung Res.

[19] Oldroyd SD, Thomas GL, Gabbiani G (1999). Interferon-gamma inhibits experimental renal fibrosis. Kidney Int.

[20] Ramos G, Hofmann U, Frantz S (2016). Myocardial fibrosis seen through the lenses of T-cell biology. J Mol Cell Cardiol.

[21] Molawi K, Wolf Y, Kandalla PK (2014). Progressive replacement of embryo-derived cardiac macrophages with age. J Exp Med.

[22] Pinto AR, Paolicelli R, Salimova E (2012). An abundant tissue macrophage population in the adult murine heart with a distinct alternatively-activated macrophage profile. PLoS ONE.

[23] National Academies Press (US) (2011). Guide for the care and use of laboratory animals.

[24] Kilkenny C, Browne WJ, Cuthill IC (2010). Improving bioscience research reporting: the ARRIVE guidelines for reporting animal research. PLoS Biol.

[25] Gergs U, Bernhardt G, Buchwalow IB (2019). Initial characterization of transgenic mice overexpressing human histamine H2 receptors. J Pharmacol Exp Ther.

[26] Boknik P, Drzewiecki K, Eskandar J (2018). Phenotyping of mice with heart specific overexpression of A2A-adenosine receptors: evidence for cardioprotective effects of A2A-adenosine receptors. Front Pharmacol.

[27] Gergs U, Jahn T, Werner F (2019). Overexpression of protein phosphatase 5 in the mouse heart: reduced contractility but increased stress tolerance - Two sides of the same coin?. PLoS ONE.

[28] Langendorff O (1895). Untersuchungen am überlebenden Säugethierherzen. Pflügers Arch.

[29] Ramos GC, van den Berg A, Nunes-Silva V (2017). Myocardial aging as a T-cell-mediated phenomenon. Proc Natl Acad Sci USA.

[30] Gergs U, Baumann M, Böckler A (2010). Cardiac overexpression of the human 5-HT4 receptor in mice. Am J Physiol Heart Circ Physiol.

[31] Bollmann P, Werner F, Jaron M (2020). Initial characterization of stressed transgenic mice with cardiomyocyte-specific overexpression of protein phosphatase 2C. Front Pharmacol.

[32] Neumann J, Bock P, Fischer M (2010). Phenotyping of heterozygous calsequestrin targeted mice. Basic Clin Pharmacol Toxicol.

[33] Song L, Alcalai R, Arad M (2007). Calsequestrin 2 (CASQ2) mutations increase expression of calreticulin and ryanodine receptors, causing catecholaminergic polymorphic ventricular tachycardia. J Clin Invest.

[34] Bers DM (2008). Calcium cycling and signaling in cardiac myocytes. Annu Rev Physiol.

[35] Chopra N, Kannankeril PJ, Yang T (2007). Modest reductions of cardiac calsequestrin increase sarcoplasmic reticulum Ca2+ leak independent of luminal Ca2+ and trigger ventricular arrhythmias in mice. Circ Res.

[36] Fallavollita JA, Lim H, Canty JJM (2001). Myocyte apoptosis and reduced SR gene expression precede the transition from chronically stunned to hibernating myocardium. J Mol Cell Cardiol.

[37] Lüss H, Meissner A, Rolf N (2000). Biochemical mechanism(s) of stunning in conscious dogs. Am J Physiol Heart Circ Physiol.

[38] Lüss H, Boknıék P, Heusch G (1998). Expression of calcium regulatory proteins in short-term hibernation and stunning in the in situ porcine heart1. Cardiovasc Res.

[39] Kim DK, Choi E, Song B-W (2012). Differentially regulated functional gene clusters identified in early hypoxic cardiomyocytes. Mol Biol Rep.

[40] Temsah RM, Kawabata K, Chapman D (2001). Modulation of cardiac sarcoplasmic reticulum gene expression by lack of oxygen and glucose. FASEB J.

[41] Goswami SK, Ranjan P, Dutta RK (2021). Management of inflammation in cardiovascular diseases. Pharmacol Res.

[42] Ma Y, Chiao YA, Clark R (2015). Deriving a cardiac ageing signature to reveal MMP-9-dependent inflammatory signalling in senescence. Cardiovasc Res.

[43] Al-Ansari F, Lahooti H, Stokes L (2018). Correlation between thyroidal and peripheral blood total T cells, CD8+ T cells, and CD8+ T- regulatory cells and T-cell reactivity to calsequestrin and collagen XIII in patients with Graves' ophthalmopathy. Endocr Res.

[44] Nguyen B, Gopinath B, Tani J (2008). Peripheral blood T lymphocyte sensitisation against calsequestrin and flavoprotein in patients with Graves' ophthalmopathy. Autoimmunity.

