# Supplementary material for: Effects of acute ischemia and hypoxia in young and adult calsequestrin (CSQ2) knock-out and wild-type mice
Source: Mol Cell Biochem. 2022 Mar 21;477(6):1789–801. doi: 10.1007/s11010-022-04407-2 (PMC9068673; doi:10.1007/s11010-022-04407-2)
Supplement: Supplementary file 1 — Supplementary file1 (DOCX 25 kb) [file 11010_2022_4407_MOESM1_ESM.docx]

**Table S1** Echocardiographic measurements in anesthetized mice. Genotype indicates CSQ2(+/+) and CSQ2(-/-) mice. Ctr means control condition that is before β-adrenergic stimulation by injection of isoprenaline. Iso means values after injection of isoprenaline. Tissue E´/A´ indicates tissue Doppler data where the E´-value is divided by the A´- value. Old means mice about 18 months old and young means about six months old. See table 1 for exact ages. Numbers of animals are indicated in the last column. X = no stable echocardiographic recordings could be obtained.

| **Echocardiographic Parameters** | **Age** | **Stimulation** | **Genotype** | | | | **Numbers (n)** |
| --- | --- | --- | --- | --- | --- | --- | --- |
|  |  |  | CSQ2(+/+) | | CSQ2(-/-) | |  |
|  |  |  | Mean | SEM | Mean | SEM | Basal |
| **Aorta ascendens peak velocity (mm/s)** | Old | Ctr | 1161.99 | 59.92 | 1072.01 | 32.97 | 8/7 |
|  | Young | Ctr | 1088.48 | 107.47 | 1000.79 | 86.58 | 6/6 |
|  |  | Iso | 950.78 | 71.82 | 1086.07 | 0.00 | 3/1 |
| **Aorta ascendens VTI (mm)** | Old | Ctr | 32.68 | 1.99 | 31.14 | 2.66 | 8/7 |
|  |  | Iso | x | x | x | x | x |
|  | Young | Ctr | 33.02 | 1.86 | 32.55 | 2.23 | 6/6 |
|  |  | Iso | 28.78 | 3.24 | 30.18 | 0.00 | 3/1 |
| **Aorta descendens peak velocity (mm/s)** | Old | Ctr | 950.61 | 28.31 | 906.12 | 60.21 | 8/7 |
|  |  | Iso | 1017.67 | 58.26 | 1144.29^+#^ | 49.97 | 3/4 |
|  | Young | Ctr | 920.36 | 34.46 | 960.36 | 57.97 | 6/7 |
|  |  | Iso | x | x | 880.15 | 23.90 | 0/2 |
| **Aorta descendens VTI (mm)** | Old | Ctr | 29.27 | 1.35 | 27.18^#^ | 1.30 | 8/7 |
|  |  | Iso | 25.41 | 2.03 | 25.91 | 1.65 | 3/4 |
|  | Young | Ctr | 29.77 | 1.71 | 32.23 | 1.68 | 6/7 |
|  |  | Iso | x | x | 28.09 | 0.98 | 0/2 |
| **Pulmonary artery peak velocity (mm/s)** | Old | Ctr | 711.40 | 31.94 | 591.18 | 56.14 | 8/7 |
|  |  | Iso | 845.89 | 57.70 | 879.39^+^ | 29.06 | 3/4 |
|  | Young | Ctr | 794.85 | 28.79 | 688.91* | 32.29 | 6/7 |
|  |  | Iso | 794.98 | 49.45 | 838.00^+^ | 34.40 | 6/7 |
| **Pulmonary artery VTI (mm)** | Old | Ctr | 25.79^ | 0.67 | 20.68*^#^ | 1.08 | 8/7 |
|  |  | Iso | 22.20 | 2.54 | 23.89 | 0.85 | 3/4 |
|  | Young | Ctr | 29.92 | 1.08 | 26.30 | 1.10 | 6/7 |
|  |  | Iso | 25.17 | 1.69 | 25.40 | 1.15 | 6/7 |
| **Mitral valve E/A** | Old | Ctr | 1.38 | 0.07 | 1.33 | 0.09 | 7/6 |
|  |  | Iso | x | x | x | x | x |
|  | Young | Ctr | 1.45 | 0.20 | 1.49 | 0.13 | 4/5 |
|  |  | Iso | x | x | x | x | x |
| **Mitral valve/Tissue E/E'** | Old | Ctr | 23.00 | 1.98 | 31.60 | 7.16 | 7/6 |
|  |  | Iso | x | x | x | x | x |
|  | Young | Ctr | 39.00 | 10.40 | 28.20 | 5.72 | 3/5 |
|  |  | Iso | x | x | x | x | x |
| **Tissue E'/A'** | Old | Ctr | 1.34 | 0.16 | 0.96 | 0.17 | 8/7 |
|  |  | Iso | 1.18 | 0.11 | 1.20 | 0.23 | 8/7 |
|  | Young | Ctr | 1.24 | 0.19 | 1.01 | 0.10 | 5/6 |
|  |  | Iso | 1.09 | 0.23 | 1.14 | 0.07 | 6/7 |
| **Ejection fraction** | Old | Ctr | 76.46 | 1.99 | 62.51* | 4.26 | 8/7 |
|  |  | Iso | 90.82^+^ | 1.61 | 71.75*^#^ | 2.89 | 8/7 |
|  | Young | Ctr | 78.77 | 1.85 | 74.50 | 4.15 | 6/7 |
|  |  | Iso | 90.75^+^ | 2.62 | 89.51^+^ | 2.60 | 6/7 |
| **Heart rate (BPM)** | Old | Ctr | 496.72 | 9.79 | 432.90* | 32.07 | 8/7 |
|  |  | Iso | 550.56^+^ | 4.41 | 532.40^+^ | 14.17 | 8/7 |
|  | Young | Ctr | 502.26 | 26.66 | 421.65 | 30.68 | 6/7 |
|  |  | Iso | 557.75 | 34.41 | 554.81^+^ | 16.86 | 6/7 |
| **Left ventricular enddiastolic diameter (mm)** | Old | Ctr | 3.22 | 0.16 | 3.31 | 0.13 | 8/6 |
|  |  | Iso | 2.78 | 0.12 | 2.90^+^ | 0.10 | 6/6 |
|  | Young | Ctr | 2.99 | 0.11 | 3.15 | 0.19 | 6/7 |
|  |  | Iso | 2.54 | 0.21 | 2.54 | 0.21 | 6/7 |
| **Left ventricular endsystolic diameter (mm)** | Old | Ctr | 1.62 | 0.05 | 2.18* | 0.13 | 6/7 |
|  |  | Iso | 0.96^+^ | 0.09 | 1.59*^+#^ | 0.11 | 6/7 |
|  | Young | Ctr | 1.63 | 0.07 | 1.82 | 0.20 | 6/7 |
|  |  | Iso | 0.95^+^ | 0.16 | 1.05^+^ | 0.17 | 6/7 |
| **Septal thickness enddiastolic (mm)** | Old | Ctr | 1.29 | 0.13 | 1.25 | 0.14 | 8/7 |
|  |  | Iso | 1.33 | 0.15 | 1.41 | 0.09 | 8/7 |
|  | Young | Ctr | 1.09 | 0.07 | 1.31 | 0.14 | 6/7 |
|  |  | Iso | 1.21 | 0.05 | 1.47 | 0.13 | 6/7 |
| **Septal thickness endsystolic (mm)** | Old | Ctr | 1.87 | 0.14 | 1.72 | 0.15 | 8/7 |
|  |  | Iso | 2.16 | 0.15 | 2.01 | 0.09 | 8/7 |
|  | Young | Ctr | 1.73 | 0.07 | 1.83 | 0.13 | 6/7 |
|  |  | Iso | 1.99 | 0.13 | 2.14 | 0.11 | 6/7 |

*p < 0.05 vs. CSQ2(+/+); ^#^p < 0.05 vs. young age group of each genotype; ^+^p < 0.05 vs. control (Ctr)

**Table S2** Strain parameters. Using echocardiography, the strain of regions of the heart assessed by B-mode measurements using the software supplied for the visual sonics system Vevo 2100. Genotype indicates CSQ2(+/+) and CSQ2(-/-) mice. Basal means control condition that is before β-adrenergic stimulation by injection of isoprenaline. Old means mice about 18 months old and young means about six months old. See table 1 for exact ages. Numbers of animals are indicated in the last column.

| **Echocardiographic Parameters** | **Age** | **Genotype** | | | | **Numbers (n)** |
| --- | --- | --- | --- | --- | --- | --- |
|  |  | CSQ2(+/+) | | CSQ2(-/-) | |  |
|  |  | Mean | SEM | Mean | SEM | Basal |
| **Radial velocity posterior mid (cm/s)** | Old | 2.05 | 0.23 | 1.83 | 0.2 | 8/8 |
|  | Young | 1.67 | 0.19 | 1.36 | 0.17 | 6/7 |
| **Radial velocity lateral wall (cm/s)** | Old | 1.54 | 0.16 | 1.05 | 0.2 | 4/4 |
|  | Young | 1.25 | 0.14 | 1.18 | 0.11 | 6/7 |
| **Longitudinal velocity posterior mid (cm/s)** | Old | 1.17 | 0.09 | 0.98 | 0.18 | 8/8 |
|  | Young | 1.49 | 0.30 | 1.00 | 0.19 | 6/7 |
| **Circumferential velocity lateral wall (deg/s)** | Old | 175.5 | 20.06 | 303.26 | 58.87 | 4/4 |
|  | Young | 176.42 | 31.05 | 213.27 | 34.64 | 6/7 |
| **Radial displacement posterior mid (mm)** | Old | 0.6 | 0.05 | 0.52 | 0.05 | 8/8 |
|  | Young | 0.52 | 0.05 | 0.42 | 0.08 | 6/7 |
| **Radial displacement lateral wall (mm)** | Old | 0.42 | 0.06 | 0.36 | 0.05 | 4/4 |
|  | Young | 0.44 | 0.06 | 0.39 | 0.02 | 6/7 |
| **Longitudinal displacement posterior mid (mm)** | Old | 0.25 | 0.03 | 0.17 | 0.02 | 8/8 |
|  | Young | 0.38 | 0.07 | 0.26 | 0.07 | 6/7 |
| **Circumferential displacement lateral wall (deg)** | Old | 3.53 | 1.06 | 5.76 | 2.1 | 4/4 |
|  | Young | 1.60 | 0.77 | 4.16 | 1.37 | 6/7 |
| **Strain radial posterior mid (%)** | Old | 23.59 | 3.24 | 25.14 | 3.42 | 8/8 |
|  | Young | 35.78 | 5.85 | 36.85 | 6.79 | 6/7 |
| **Strain radial lateral wall (%)** | Old | 36.88^#^ | 3.07 | 32.91^#^ | 8.22 | 4/4 |
|  | Young | 14.54 | 4.41 | 11.21 | 2.31 | 6/7 |
| **Strain longitudinal posterior mid (%)** | Old | 13.15^#^ | 1.33 | 16.5^#^ | 1.74 | 8/8 |
|  | Young | 22.20 | 2.63 | 10.56* | 1.79 | 6/7 |
| **Strain circumferential lateral wall (%)** | Old | 30.64 | 5.08 | 31.38 | 7.68 | 4/4 |
|  | Young | 27.28 | 2.23 | 28.12 | 2.10 | 6/7 |
| **Strain rate radial posterior mid (1/s)** | Old | 8.89 | 0.84 | 8.3 | 1.21 | 8/8 |
|  | Young | 10.35 | 1.45 | 8.89 | 0.93 | 6/7 |
| **Strain rate radial lateral wall (1/s)** | Old | 10.34 | 0.9 | 8.74 | 1.72 | 4/4 |
|  | Young | 7.89 | 1.27 | 7.90 | 1.22 | 6/7 |
| **Strain rate longitudinal posterior mid (1/s)** | Old | 6.86 | 0.72 | 7.49^#^ | 0.87 | 8/8 |
|  | Young | 8.46 | 1.15 | 4.87* | 0.41 | 6/7 |
| **Strain rate circumferential lateral wall (1/s)** | Old | 16.14 | 3.52 | 4.12 | 3.82 | 4/4 |
|  | Young | 10.88 | 0.67 | 11.33 | 1.18 | 6/7 |

*p < 0.05 vs. CSQ2(+/+); ^#^p < 0.05 vs. young age group of each genotype
